# Design and Characterization of a Microfluidic Circuit for Air Particulate Matter Separation

**DOI:** 10.3390/mi13020252

**Published:** 2022-02-02

**Authors:** Yongzhen Li, Yaru Xu, Jinling Jiang, Xiaofeng Zhu, Ruihua Guo, Jianhai Sun

**Affiliations:** 1 Institute of Urban Safety and Environmental Science, Beijing Academy of Science and Technology, Beijing 100054, China; liyongzhen@bmilp.com (Y.L.); zxf_402@163.com (X.Z.); 2 College of Robotics, Beijing Union University, Beijing 100020, China; jqryaru@buu.edu.cn; 3 Beijing Aerospace Measure and Control Corp (AMC) Ltd., Beijing 100041, China; jiangjinling45@126.com; 4 Aerospace Information Research Institute, Chinese Academy of Sciences, Beijing 100194, China

**Keywords:** particulate matter, air microfluidic circuit, collection efficiency, particle wall loss

## Abstract

Air microfluidic circuits have been widely concerned in the separation of atmospheric particulate matter, especially for portable particulate matter separation detection devices. Currently, no systematic approach for the design and optimization of an air-microfluidic system for PM separation has been reported in the literature. In this paper, a two-stage air microfluidic circuit is designed. The design process is divided into two stages: first, the preliminary design of the structure is completed according to aerodynamic theory. Then, the influences of various factors (such as flow channel width, tilt angle, flow rate, etc.) on the collection efficiency and particle wall loss are explored through numerical analysis to complete the optimization design of the structure. Finally, the air microfluidic circuit is prepared by MEMS processing technology and the particulate matter separation experiments are carried out. The developed two-stage air microfluidic circuit can realize the efficient separation of PM10 and PM2.5. Thus, the important factors affecting the collection efficiency and particle wall loss of air microfluidic circuit are clarified, and a systematic design theory method is formed.

## 1. Introduction

The problem of airborne particulate pollution is becoming more and more serious. The particulate matters (PMs) are mainly composed of solid and liquid particles suspended in the air which carry a large number of viruses and bacteria. According to the different aerodynamic diameter (AD), PMs can be divided into coarse particles (particles with AD greater than 2.5 μm), fine particles (particles with AD less than 2.5 μm), and ultrafine particles (particles with AD less than 100 nm) [1,2,3]. In general, the particulate matters of 10 μm, 2.5 μm, and even 1 μm are getting more attention. Long-term exposure to high concentrations of PMs is very harmful to the human body, especially concerning fine particles. Due to their small particle size, fine particles can reach deep into the respiratory system, resulting in a variety of diseases, such as asthma, lung cancer, and cardiopulmonary mortality [4,5,6,7]. Compared with fine particles, ultrafine particles do more serious harm to human health, as they can penetrate the pulmonary and cardiovascular systems and give rise to lasting conditions, such as increased predisposition to heart diseases or premature births and affect fetal development [8,9]. Therefore, it is more and more urgent to identify the composition characteristics of PMs. The premise of this work is to achieve the separation of PMs of different sizes. Then, the analysis of the composition of different particle sizes can provide scientific guidance for the treatment of airborne particulate pollution.

**Figure 1:**
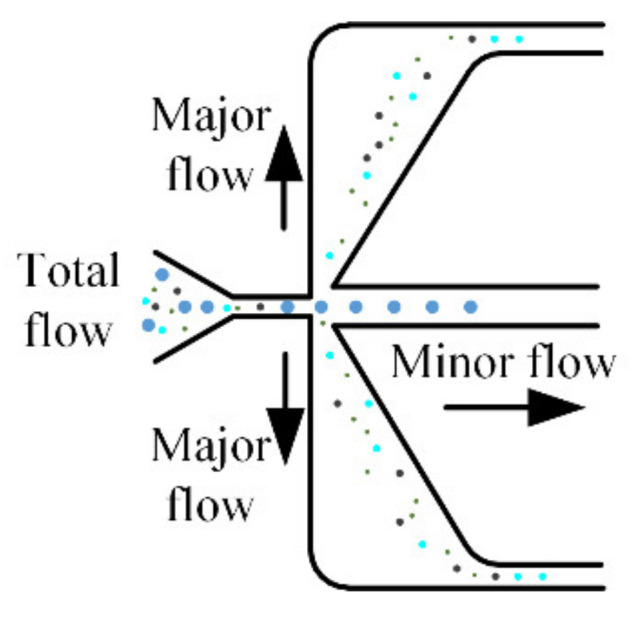
Particle separation process of the air microfluidic circuit.

The separation of particles with different particle sizes can be achieved by aerodynamic [10], electrostatic [11,12,13], and thermophoretic techniques [14,15]. Among a variety of atmospheric particle size separation methods, the virtual or inertial impactor based on the aerodynamics theory has been favored by many scholars due to its miniaturization, high separation efficiency, and simple structure [10,16,17]. In order to achieve a small footprint and portable equipment, the air microfluidic circuit based on the virtual impactor was born [18,19]. The air microfluidic circuit realizes the separation of desired particles by the principle of different inertial forces. Since then, there have been many types of miniaturized air microfluidic circuits [1,20,21,22,23,24,25,26,27,28]. In addition, many virtual impactors are relatively large [29,30,31,32]. The basic working principle of these devices is shown in Figure 1.

According to aerodynamic theory, the particles with greater inertia enter the minor flow channels, while the particles with smaller inertia remain in the major flow. During this process, some particles may collide with the nozzle or the inner wall of the flow channel and be lost. Therefore, particle wall loss is as important as collection efficiency in evaluating the performance of microfluidic circuits. A good microfluidic circuit should have a sharp separation curve with little wall loss. In an effort to achieve the above goals, it has become a central research topic to explore the key factors affecting the two performances of microfluidic circuits. Some special structural designs are summarized in [33]. Lee [16] focused on the effect of an orifice on collection efficiency and wall loss of a slit virtual impactor. Lim [17] evaluated the collection efficiency and particle wall losses of three types of virtual impactors with different pressure drops. Zahir [34,35] explored the effects of different structural forms on the collection efficiency and particle wall loss of virtual impactors. It was proved that the Stokes number and wall loss can be reduced by using the three-partitioned horizontal inlet. The design of the air microfluidic circuit then becomes the most critical step involving the selection of many parameters. The influence of structural parameters on device performance is less involved. Chen [36] only studied the effects of some parameters on collection efficiency but did not involve the performance of wall loss. It is not known how to determine the parameters in the structural design of microfluidic circuits. This is also the main reason for carrying out this study.

**Figure 2:**
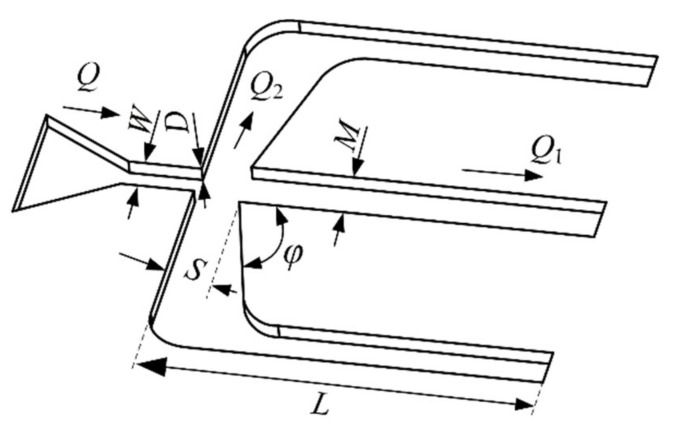
Structure parameter definitions of the air microfluidic circuit.

The specific structure parameter definitions of the air microfluidic circuit are shown in Figure 2. In the design of microfluidic circuits, the cutoff diameter of particles to be separated should be determined first. The cutoff diameter is usually defined as the particle diameter corresponding to the collection efficiency equal to 50%, and the specific expression can be approximated as [10]

(1) $$d_{50}=\sqrt{\frac{9\eta W^{2}D\left(Stk_{50}\right)}{\rho QC_{c}}}$$

where *η* is the dynamic viscosity of air; *W* and *D* are the width and depth of the impactor jet, respectively; *ρ* is the particle density; *Q* is the volumetric flow rate through the inlet jet; *Stk*_50_ is the Stokes number, which is recommended to be 0.59 for rectangular jet impactors [37]; and *C_c_* is the Cunnigham correction factor, for particles larger than 1μm; it can be given as

(2) $$C_{c}=1+\frac{2.52\lambda }{d}$$

where *d* is the particle diameter and *λ* is the length of the mean free path of the air. The *C_c_* is recommended to be 1.166 [21]. 

Equation (1) only reflects the relationship between the cut-off diameter and the width and depth of the impactor jet. The specific effects of structure parameters on the collection efficiency have not been reflected and are rarely reported [38]. In addition, most studies only focus on collection efficiency but do not pay attention to the relationship between particle wall loss and structure parameters. In this paper, a two-stage air microfluidic circuit for separating atmospheric particles is designed based on aerodynamics theory. Through numerical analysis, the influencing factors of the collection efficiency and the wall loss are explored and the structural design of the air microfluidic circuit is completed. Based on MEMS processing technology, an air microfluidic circuit is fabricated and evaluated by experiments. Thus, it is proved that the developed air microfluidic circuit can realize the effective separation of PM10 and PM2.5. It also has important reference significance for the optimization design of air microfluidic circuits.

## 2. Design of Air Microfluidic Circuit

**Figure 3:**
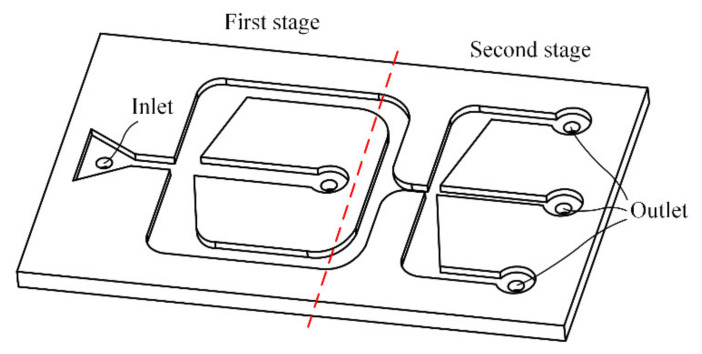
Two-stage air microfluidic circuit.

Currently, the environmental pollution of PM10 and PM2.5 has been the focus of attention. Therefore, the separations of 10 μm and 2.5 μm particles are mainly studied in this paper. That is, two air microfluidic circuits need to be cascaded, as shown in Figure 3. The first stage of separation of the PMs is carried out; the particles with a size greater than 10 μm move into the minor flow channel along a straight-line while the other particles flow into the major flow channel. Then, the second stage is implemented for particles less than 2.5 μm. Therefore, the two-stage microfluidic circuit has three exits. The particles larger than 10 μm can be separated by the minor flow channel of the first stage and particles of 2.5 μm to 10 μm are separated at the exit of the minor flow channel. At the outlet of the major flow channel of the second stage, particles less than 2.5 μm are separated. An air sampler is placed at the outlet of the air microfluidic circuit to provide airflow. 

The structural design of the two-stage air microfluidic circuit cannot be realized only according to Equations (1) and (2). The key factors affecting the collection efficiency and particle wall loss will be explored by numerical analysis method as well to assist the systematic design of air microfluidic circuits. The collection efficiency (*η*) and wall loss (WL) are calculated as [16]

(3) $$\eta =\frac{N_{M}}{N_{M}+N_{m}}$$

(4) $$\mathrm{WL}=1-\frac{N_{M}+N_{m}}{N_{\mathrm{in}}}$$

where *N*_M_ is the number of particles collected in the major channel, *N*_m_ is the number of the particles collected in the minor channel, and *N*_in_ is the number of the particles entering the microfluidic circuit.

## 3. Numerical Analysis

In order to realize the structural design of the air microfluidic circuit, a numerical analysis is implemented using the software COMSOL, which is the advocator and leader of multi-physical field modeling and simulation. The separation process of PMs involves an analysis of flow field and particle trajectory, and the COMSOL happens to have a microfluidic module and a particle tracking module. Due to the small size of the air microfluidic circuit and the small Reynolds number of air flowing in the circuit, the airflow can be regarded as laminar flow. Although the PMs in the air are irregular, they are assumed to be spherical during the simulation for convenience. In addition, some of the other parameters are shown in Table 1.

**Figure 4:**
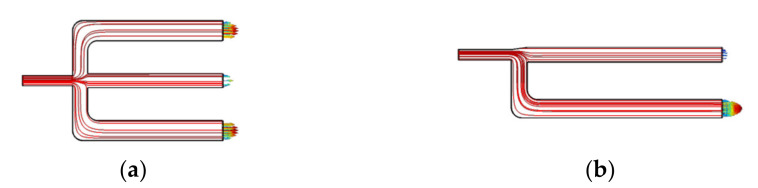
The trajectory of particles. (**a**) Symmetry style. (**b**) Asymmetry style.

In the process of simulation, the flow field of the air microfluidic circuit is calculated first and then the process of separating the particles is carried out. These two processes are carried out separately and independently, which can effectively reduce the calculation time and improve work efficiency. When the PMs with different attributes are separated, there is no need to calculate the flow field, only the particle separation process needs to be performed. The trajectory of particles in the separation process of PMs is shown in Figure 4.

### 3.1. Analysis of the Influence of Structural Style

**Figure 5:**
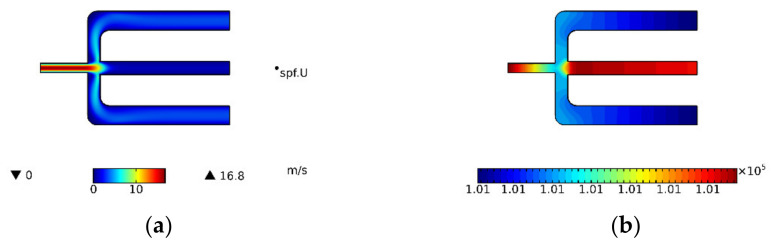
Symmetry style. (**a**) Air velocity and (**b**) Pressure distribution.

**Figure 6:**
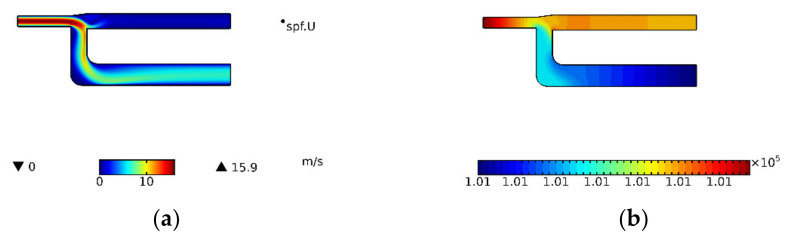
Asymmetry style. (**a**) Air velocity and (**b**) Pressure distribution.

It is necessary to determine the structural style of the air microfluidic circuit as symmetric or asymmetric in structural style. The air velocity and pressure distributions in the symmetrical and asymmetric air microfluidic circuit are shown in Figure 5 and Figure 6. These two performances can be considered the same. That is, the two structure styles can provide the same aerodynamics for PMs. The selection of structural style also needs to refer to the separation effect of particles and the results of particle wall loss. 

**Figure 7:**
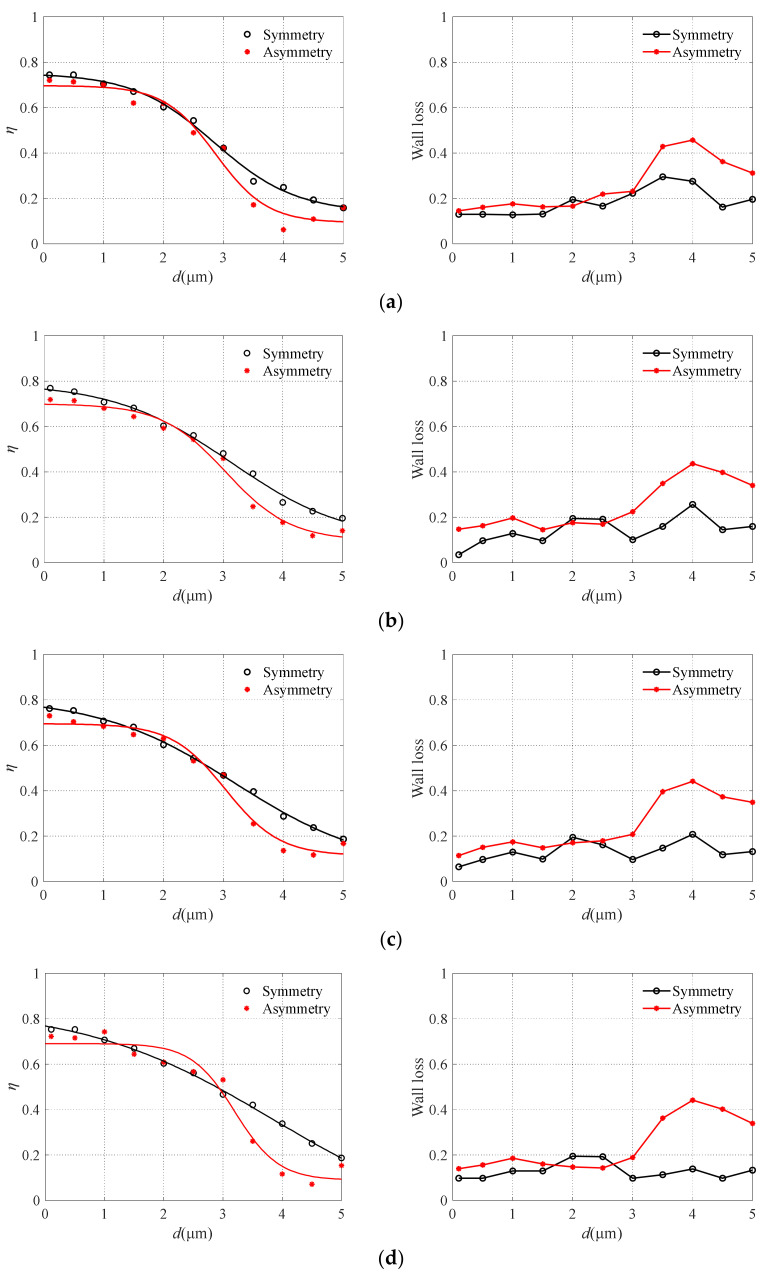
The particle wall loss with different structural style. (**a**) *S* = 260 μm, (**b**) *S* = 300 μm, (**c**) *S* = 340 μm, and (**d**) *S* = 380 μm.

The two performances of the collection efficiency and particle wall loss of symmetrical and asymmetric virtual impactors are compared and the results are shown in Figure 7. The smooth curves in Figure 7 represent the fitting curve of finite element analysis data. The air microfluidic circuit with an asymmetric structure can obtain a steeper collection efficiency curve. Under the same conditions, symmetric structure increases the possibility of particles entering the major flow channel, thus increasing the collection efficiency leading to the inferior separation effect of particles for symmetric structure. When the particle diameter is larger than 3 μm, the wall loss greatly increases with the increase in particle size. For particles smaller than 3 μm in size, the wall loss in the circuit is not serious. Our focus is on the separation effect of particles smaller than 3 μm in microfluidic circuits, therefore, the structure design of the air microfluidic circuit can be considered as an asymmetric style.

### 3.2. Analysis of the Influence of Major Flow Channel Width S and Minor Flow Channel Width M

**Figure 8:**
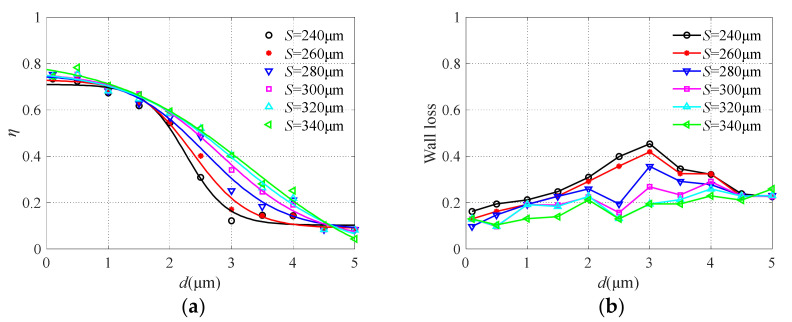
The collection efficiency curve (**a**) and wall loss (**b**) with different major flow channel widths.

The major flow channel width *S* was changed from 240 μm to 340 μm, and the results are shown in Figure 8. It can be seen that the change of major flow channel width has an obvious influence on the collection efficiency. With the increase of channel width, the sharpness of the collection efficiency curve decreases, which is not conducive to the effective separation of particulate matter. While the particle wall loss increases with the decrease of the major flow channel due to turbulence in the restricted area between the collection probe and nozzle exit, similar conclusions can also be found in [39]. Therefore, the two performance requirements of collection efficiency and particle wall loss should be considered comprehensively. For particles with large sizes, the wider the major flow channel width is, the easier it is to enter. Thus, the collection efficiency is reduced and the particles are less likely to collide with the wall. When the major flow channel width *S* = 280 μm, the collection efficiency curve is relatively steep, which can realize the effective separation of particles with a diameter of 2.5 μm, and the particle wall loss is low.

**Figure 9:**
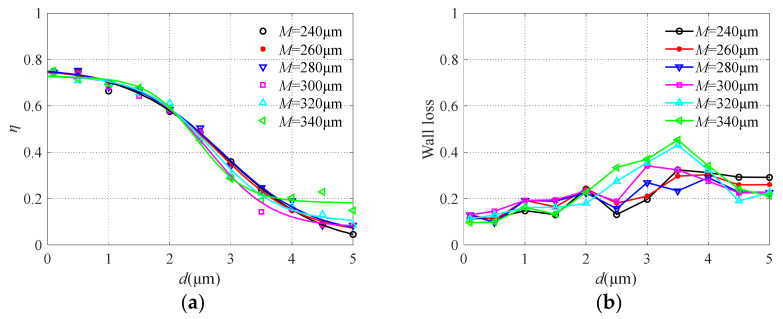
The collection efficiency curve (**a**) and wall loss (**b**) with different minor flow channel widths.

The simulation results obtained from adjusting the value of the minor flow channel width *M* (240 μm~340 μm) are shown in Figure 9. Obviously, the minor flow channel width has little effect on the particulate collection efficiency. However, the particle wall loss will increase to a certain extent with the increase of *M*. According to Equations (3) and (4), the collection efficiency is related to the particle wall loss and the number of particles entering the main channel. The variation of the width of the minor flow channel has little effect on the collection efficiency. The increase of *M* will cause the increase of particle wall loss to a certain extent. This is because the enlarged minor flow channel provides sufficient space for many streamlines to make an almost complete U-turn which results in significant losses on both the collection probe and the backside of the acceleration nozzle [40,41,42].

### 3.3. Analysis of the Influence of Tilt Angle φ

**Figure 10:**
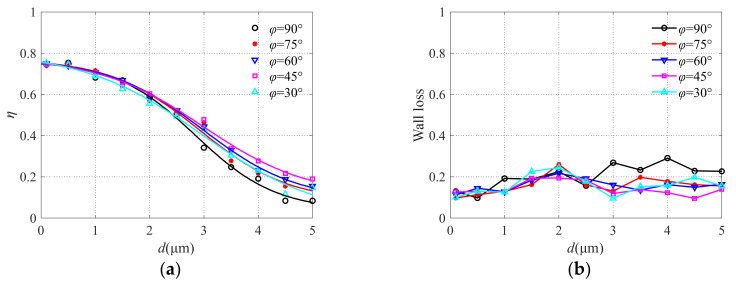
The collection efficiency curve (**a**) and wall loss (**b**) with different angles.

Different tilt angles were designed and the simulation results obtained are shown in Figure 10. The steepness of the collection efficiency curve increases with the increase of the angle *φ* in the range of 45°~90°. When *φ* = 90°, the separation effect of the virtual impactor is the best, but the particle wall loss also increases with the increase in particle size. It is not hard to imagine that when the tile angle is 90° particles easily collide with the inner wall which leads to the reduction of collection efficiency, especially for particles with large sizes. Therefore, the tilt angle is set as 75° when designing the microfluidic circuit.

### 3.4. Analysis of the Influence of Inlet Flow Q

**Figure 11:**
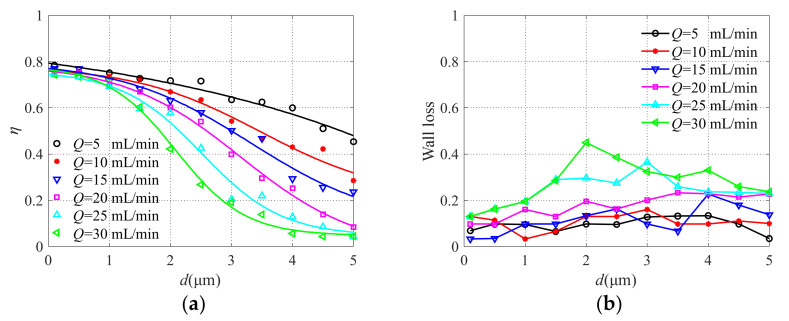
The collection efficiency curve (**a**) and wall loss (**b**) with different inlet flows.

The inlet flow changes from 5 mL/min to 30 mL/min and the simulation results obtained are shown in Figure 11. With the increase of inlet flow *Q*, the steepness of the collection efficiency curve increases, the separation effect of particles becomes better, and the cutoff diameter decreases. At the same time, the particle wall loss rate increases. According to Equation (1), the larger the inlet flow is, the smaller the cutoff diameter is, and the easier it is to separate particles with different particle sizes. Meanwhile, the probability of particle collision increases, leading to the increase of particle wall loss. Moreover, according to the expression of the Reynolds number, the Reynolds number will increase with the increase of inlet flow. The sharpness of the collection efficiency curve increases and the cutoff size decreases as the Reynolds number increases [40]. Taken together, the inlet flow is selected as 20 mL/min. The effects of *S*, *M,* and *Q* on the collection efficiency can also be found in [36].

### 3.5. Analysis of the Influence of Flow Channel Length L

**Figure 12:**
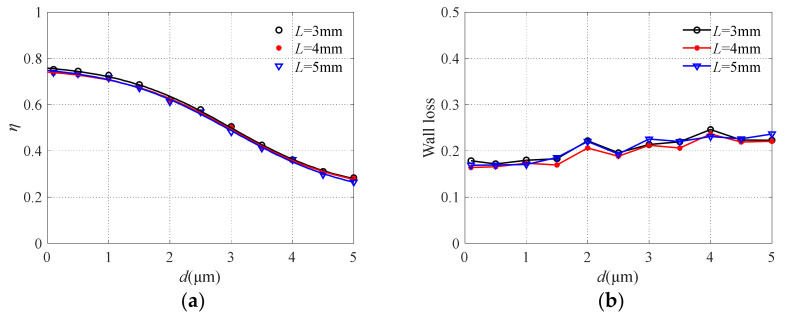
The collection efficiency curve (**a**) and wall loss (**b**) with different flow channel lengths.

The results of collection efficiency and particle wall loss obtained by changing the length of the major and minor flow channels are shown in Figure 12. It is easy to see that the influence of flow channel length on collection efficiency and particle wall loss is negligible. Since the separation of particles with different particle sizes occurs at the nozzle, the change of flow channel length only affects the particle movement time. The nozzle where the particles are accelerated has a strong effect on the sharpness of the collection efficiency curve [43].

## 4. Design and Fabrication of the Two-Stage Air Microfluidic Circuit

**Figure 13:**
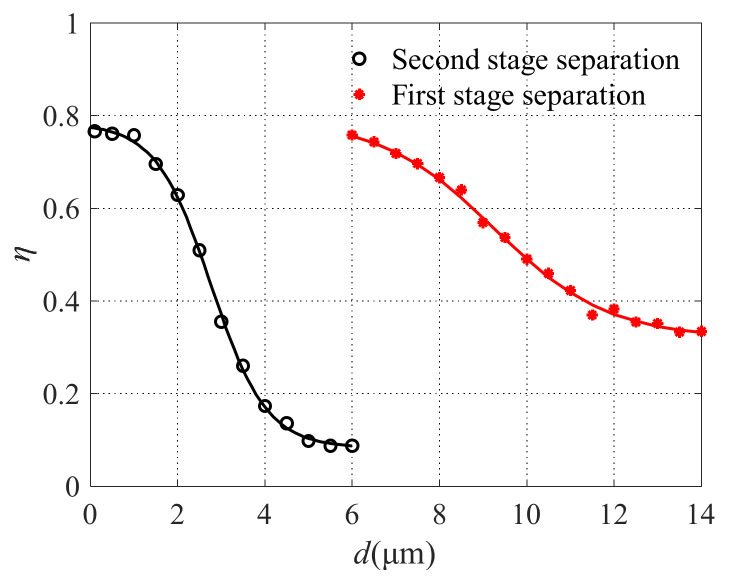
The collection efficiency curve of the two-stage microfluidic circuit.

According to the cut-off diameter expression, Equation (1), and the conclusion of numerical analysis, the design of two-stage air microfluidic circuit was realized. The structural and geometric parameters of the two-stage air microfluidic circuit are listed in Table 2. Then, finite element simulation analysis was carried out on the designed two-stage microfluidic circuit and the results shown in Figure 13. It can be seen that the microfluidic circuit can achieve effective separation of PM10 and PM2.5, thus proving the acceptability of the above analysis.

**Figure 14:**
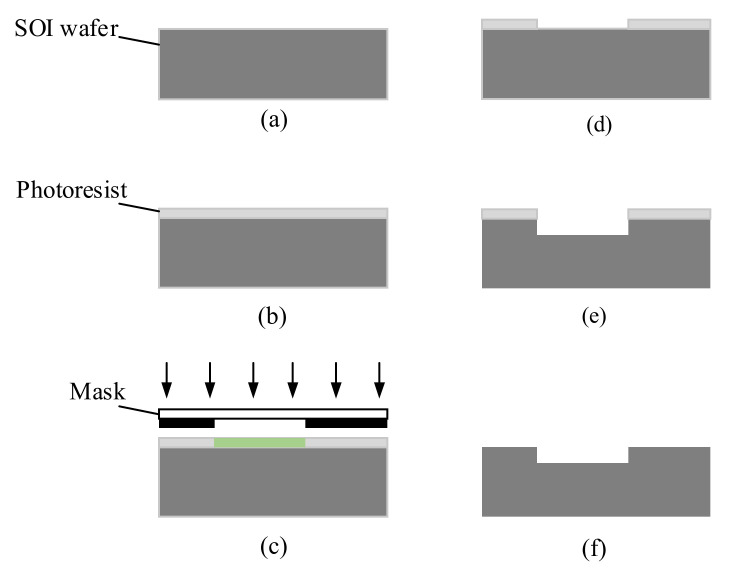
The fabrication process of the flow channel. (**a**) Preparation of SOI wafer (**b**) Coating photoresist (**c**) Exposure (**d**) Developing (**e**) Deep reactive ion etch (**f**) Stripping of photoresist.

**Figure 15:**
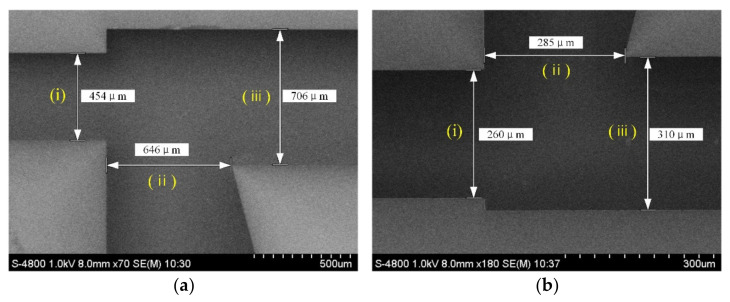
An SEM image of the microfabricated virtual impactor: (i) jet; (ii) major flow; and (iii) minor flow. (**a**) The first stage and (**b**) The second stage.

The air microfluidic circuit was then processed and prepared according to the design. The step-by-step fabrication process of the flow channel is shown in Figure 14. According to Equation (1), the cut-off diameter of atmospheric particles is affected by the depth of the flow channel in the microfluidic circuit. Silicon-on-insulator (SOI) was selected as the substrate to ensure the machining accuracy of the flow channel depth. As a substrate, the SOI wafer was etched out to provide the inlet, outlet, major flow channel, and minor flow channel. After cleaning the SOI wafer, the photoresist was deposited on its surface. The image on the mask was then imprinted onto the photoresist by exposure. The SOI wafer was developed in 1% sodium hydroxide solution. Finally, the photoresist was removed from the surface of the SOI wafer. The air microfluidic circuit was then characterized by a scanning electron microscope (SEM), as shown in Figure 15. The maximum error of the main structural dimensions was not greater than 10μm, which is approximately a 3.3% deviation from the designed value.

## 5. Experimental Evaluation

**Figure 16:**
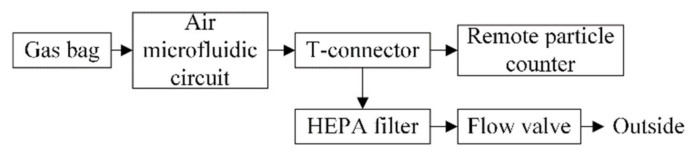
Scheme of the experimental test platform.

**Figure 17:**
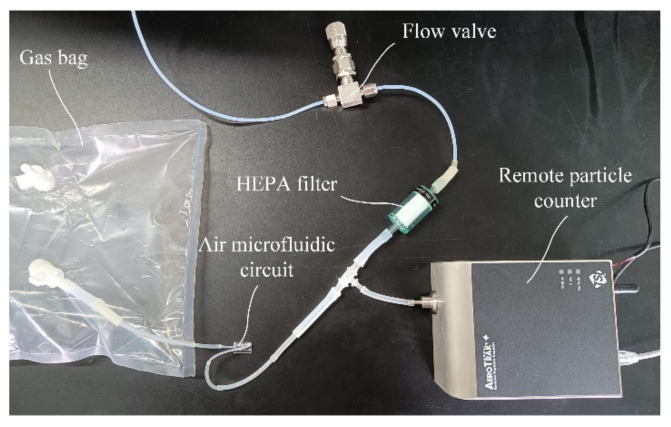
Experimental test platform.

Reference material consisting of polystyrene latex and diethyl-benzene crosslinking is used instead of atmospheric particulate matter which gives the particles good durability and physical and chemical stability. A certain amount of latex particle size reference material is injected into a 10 L gas bag with two valves and mixed thoroughly. Then, the gas bag is connected through a hose to the inlet end of the air microfluidic circuit. A remote particle counter TSI 6301 is used to record the number of particles at the outlet of the air microfluidic circuit and its flow rate was 2.83 L/min. In addition, it provided power for the particulate matter in the gas bag to be classified by the air microfluidic circuit. Since the flow required by the air microfluidic circuit is relatively small, the inlet flow of the remote particle counter was divided into two channels, one of which is connected to the outlet of the air microfluidic circuit, and the other was connected to a high efficiency particle air (HEPA) filter. The flow through the air microfluidic circuit was controlled by the flow valve at the front of the HEPA filter. In order to reduce the particle attachment in the pipeline, the entire air path should be as short as possible. The scheme and objects of the experimental setup used to characterize the performances of the air microfluidic circuit are shown in Figure 16 and Figure 17.

**Figure 18:**
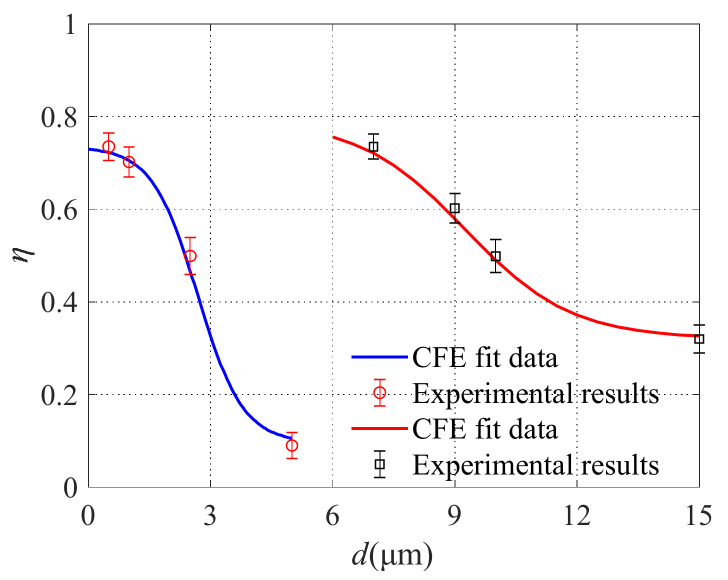
Experimental results.

Reference materials with different particle sizes were used and the specific parameters are listed in Table 3. Before sampling, the reference material was shaken well. According to the number of particles in the 10 mL reference material given in Table 3, the number of particles in the 1 mL reference material can be known. That is, the number of particles (*N*_in_) entering the microfluidic circuit was obtained. The number of particles at each outlet of the air microfluidic circuit is recorded by the remote particle counter. Furthermore, the collection efficiency of the air microfluidic circuit could be obtained. The experiment was repeated three times under the same conditions. The comparison between the FEA results and experimental results of particle separation using the designed air microfluidic circuit is shown in Figure 18. It can be seen that the experimental results of atmospheric particulate matter separation are basically consistent with the finite element simulation results, i.e., the designed microfluidic chip can effectively separate PM10 and PM2.5 particles. The results of finite element analysis can be used to guide the structural design of microfluidic circuits.

Nevertheless, the air microfluidic circuit designed in this paper is currently in the laboratory research stage which is still a long way from the market. By recording the number of particles to obtain the collection efficiency of the air microfluidic circuit, more errors will be introduced, resulting in poor accuracy of test results. Nowadays, mass-sensitive surface acoustic resonators or thin-film bulk acoustic resonators are widely used to obtain the mass concentration of particles. Moreover, they are easily integrated with the air microfluidic circuit. This will be one of the future research contents.

## 6. Conclusions

According to the aerodynamics theory, a two-stage air microfluidic circuit was preliminarily designed. Then, the collection efficiency and particle wall loss of the circuit were focused on through numerical analysis and the specific factors affecting them were explored. Analysis indicates that the sharpness of the collection efficiency curve increases with the decrease of major flow channel width, while the particle wall loss increases. The minor flow channel width has little effect on the collection efficiency, and its increase will lead to the increase of particle wall loss. When the tilt angle is 75°, a sharp collection efficiency curve and low particle wall loss can be obtained. With the increase of inlet flow, both the sharp collection efficiency curve and the particle wall loss rate increase, while the cutoff diameter decreases. The effects of the length of the major and minor flow channels on the collection efficiency and particle wall loss can be ignored. Compared with the symmetrical air microfluidic circuit, the asymmetrical structure can achieve better collection efficiency. To sum up, the asymmetric circuit structure was selected and optimized.

Based on the theoretical guidance and numerical analysis, a two-stage air microfluidic circuit was prepared and tested. It can be seen from experimental results that the air microfluidic circuit exhibits excellent measurement performances. In the future, the integrated design of the air microfluidic circuit and the mass concentration detection device will be carried out.

## Figures and Tables

**Table 1:** Key geometric parameters and material properties.

Parameters	Values
Air density *ρ*_1_ (Kg/m^3^)	1.29
Particle density *ρ*_2_ (Kg/m^3^)	1000
Dynamic viscosity *η* (Pa.s)	1.81 × 10^−5^

**Table 2:** Key geometric parameters.

Parameters	Values	Parameters	Values
*W*_1_ (μm)	450	*W*_2_ (μm)	250
*D*_1_ (μm)	200	*D*_2_ (μm)	200
*S*_1_ (μm)	640	*S*_2_ (μm)	280
*M*_1_ (μm)	700	*M*_2_ (μm)	300
*φ*_1_ (°)	75	*φ*_2_ (°)	75

**Table 3:** Specific parameters of nine kinds of reference materials.

No.	Particle Size (μm)	Uncertainty k = 2 (μm)	Number of Particles (/10 mL)
1	1.5	0.11	5.39 × 10^9^
2	2	0.2	2.27 × 10^9^
3	2.5	0.2	1.16 × 10^9^
4	3	0.3	6.74 × 10^8^
5	5	0.2	1.45 × 10^8^
6	7	0.1	5.3 × 10^8^
7	9	0.14	2.49 × 10^8^
8	10	0.2	1.82 × 10^8^
9	15	0.3	5.39 × 10^7^

## References

[1] Fahimi D., Mahdavipour O., Sabino J., White R., Paprotny I. (2019). Vertically-stacked MEMS PM 2.5 sensor for wearable applications. Sens. Actuators A Phys..

[2] Miller M.R., Raftis J.B., Langrish J.P., Mclean S.G., Samutrtai P., Connell S.P., Wilson S., Vesey A.T., Fokkens P.H.B., Boere A.J.F. (2017). Inhaled Nanoparticles Accumulate at Sites of Vascular Disease. ACS Nano.

[3] Wang Y., Wang Y.S., Chen D.Y., Liu X.X., Wu C.J., Xie J. (2018). A Miniature System for Separation and Detection of PM Based on 3D Printed Virtual Impactor and QCM Sensor. IEEE Sens. J..

[4] Kim K.H., Kabir E., Kabir S. (2015). A review on the human health impact of airborne particulate matter—ScienceDirect. Environ. Int..

[5] Bowe B., Yan X., Li T., Yan Y., Xian H., Al-Aly Z. (2017). Particulate Matter Air Pollution and the Risk of Incident CKD and Progression to ESRD. J. Am. Soc. Nephrol..

[6] Basu R., Harris M., Sie L., Malig B., Broadwin R., Green R. (2014). Effects of fine particulate matter and its constituents on low birth weight among full-term infants in California. Environ. Res. Sect. A.

[7] Buggiano V., Petrillo E., Allo M., Lafaille C., Redal M.A., Alghamdi M.A., Khoder M.I., Shamy M., Munoz M.J., Kornblihtt A.R. (2015). Effects of airborne particulate matter on alternative pre-mRNA splicing in colon cancer cells. Environ. Res..

[8] Kampa M., Castanas E. (2008). Human health effects of air pollution. Environ. Pollut..

[9] Seaton A., Godden D., Macnee W., Donaldson K. (1995). Particulate air pollution and acute health effects. Lancet.

[10] Poenar D.P. (2019). Microfluidic and Micromachined/MEMS Devices for Separation, Discrimination and Detection of Airborne Particles for Pollution Monitoring. Micromachines.

[11] Bertke M., Kirsch I., Uhde E., Peiner E. (2021). Ultrafine Aerosol Particle Sizer Based on Piezoresistive Microcantilever Resonators with Integrated Air-Flow Channel. Sensors.

[12] Critelli I., Tasora A., Degiorgi A., Colledani M. Particle Simulation of Granular Flows in Electrostatic Separation Processes. Proceedings of the Sixth International Conference on Advances in System Simulation.

[13] Kawamoto H. (2008). Some techniques on electrostatic separation of particle size utilizing electrostatic traveling-wave field. J. Electrost..

[14] Eslamian M., Saghir M.Z. (2013). Novel thermophoretic particle separators: Numerical analysis and simulation. Appl. Therm. Eng..

[15] Geelhoed P.F., Lindken R., Westerweel J. (2006). Thermophoretic Separation in Microfluidics. Chem. Eng. Res. Des..

[16] Lee H., Jo D.H., Kim W.G., Yook S.J., Ahn K.H. (2014). Effect of an Orifice on Collection Efficiency and Wall Loss of a Slit Virtual Impactor. Aerosol Sci. Technol..

[17] Lim K.S., Lee K.W. (2006). Collection efficiency and particle loss of virtual impactors with different methods of increasing pressure drop. J. Aerosol Sci..

[18] Kim Y.H., Maeng J.Y., Park D., Jung I.H., Hwang J., Kim Y.J. (2007). Micromachined cascade virtual impactor with a flow rate distributor for wide range airborne particle classification. Appl. Phys. Lett..

[19] Paprotny I., Doering F., White R.M. (2010). MEMS Particulate Matter (PM) monitor for cellular deployment. IEEE Sens..

[20] Li Y., Pang W., Sun C., Zhou Q., Lin Z.Z., Chang Y., Li Q.N., Zhang M.L., Duan X.X. (2019). Smartphone-Enabled Aerosol Particle Analysis Device. IEEE Access.

[21] Wang Y., Wang Y.S., Liu W.X., Chen D.Y., Wu C.J., Xie J. (2019). An aerosol sensor for PM1 concentration detection based on 3D printed virtual impactor and SAW sensor. Sens. Actuators A Phys..

[22] Liu J.L., Hao W.C., Liu M.H., Liang Y., He S.T. (2018). A Novel Particulate Matter 2.5 Sensor Based on Surface Acoustic Wave Technology. Appl. Sci..

[23] Kim H.L., Han J., Lee S.M., Kwon H.B., Hwang J. (2018). MEMS-based particle detection system for measuring airborne ultrafine particles. Sens. Actuators A Phys..

[24] Don M., Iervolino E., Santagata F., Zhang G.Y., Zhang G.Q. (2017). Integrated Virtual Impactor Enabled PM2.5 Sensor. IEEE Sens. J..

[25] Luo Y., Sheng Y., Jiao Z., Deng Y. Microfluidic system for fine particulate matter separation and sampling. Proceedings of the 13th International Conference on Ubiquitous Robots and Ambient Intelligence (URAI).

[26] Fahimi D., Mahdavipour O., Cados T., Kirchstetter T., Solomon P., Gundel L., White R.M., Fukushima N., Nagai H., Saitoh M. MEMS Air-Microfluidic Lab-on-a-Chip Sensor for Personal Monitoring of Airborne Particulate Matter (PM2.5). Proceedings of the Hilton Head Workshop: A Solid-State Sensors.

[27] Sun J.W., Yang K., Liu Z.W., Lu Y.W. A system of continuous particles monitoring using virtual impactor. Proceedings of the 12th IEEE International Conference on Electronic Measurement & Instruments (ICEMI), IEEE.

[28] Paprotny I., Doering F., Solomon P.A., White R.M., Gundel L.A. (2013). Microfabricated air-microfluidic sensor for personal monitoring of airborne particulate matter: Design, fabrication, and experimental results. Sens. Actuators A Phys..

[29] Kim H.L., Han J.S., Lee S.M., Kwon H.B., Hwang J., Kim Y.J. Ultrafine particle counter using a MEMS-based particle processing chip. Proceedings of the 2015 28th IEEE International Conference on Micro Electro Mechanical Systems (MEMS).

[30] Liang D., Shih W.P., Chen C.S., Dai C.A. (2010). A Miniature System for Separating Aerosol Particles and Measuring Mass Concentrations. Sensors.

[31] Metcalf A.R., Narayan S., Dutcher C.S. (2018). A review of microfluidic concepts and applications for atmospheric aerosol science. Aerosol Sci. Technol. J. Am. Assoc. Aerosol Res..

[32] Zhao J.X., Liu M.L., Liang L., Wang W., Xie J. (2016). Airborne particulate matter classification and concentration detection based on 3D printed virtual impactor and quartz crystal microbalance sensor. Sens. Actuators A Phys..

[33] Le T.C., Tsai C.J. (2021). Inertial Impaction Technique for the Classification of Particulate Matters and Nanoparticles: A Review. Powder Part..

[34] Zahir M.Z., Heo J.E., Yook S.J. (2018). Effects of Three-partitioned Horizontal Inlet and Clean Air on Collection Efficiency and Wall Loss of Slit Virtual Impactors. Aerosol Air Qual. Res..

[35] Zahir M.Z., Heo J.E., Yook S.J. (2019). Influence of clean air and inlet configuration on the performance of slit nozzle virtual impactor. Adv. Powder Technol..

[36] Chen T.T., Sun J.H., Ma T.J., Li T., Liu C., Zhu X.F., Xue N. (2019). Design and Analysis of Particulate Matter Air-Microfluidic Grading Chip Based on MEMS. Micromachines.

[37] Laucks M.L. (2000). Aerosol Technology Properties, Behavior, and Measurement of Airborne Particles. J. Aerosol Sci..

[38] Chang P.K., Hsiao T.C., Engling G., Chen J.C. (2019). Computational fluid dynamics study of the effects of flow and geometry parameters on a linear-slit virtual impactor for sampling and concentrating aerosols. J. Aerosol Sci..

[39] Marple V.A., Chien C.M. (1980). Virtual impactors: A theoretical study. Environ. Sci. Technol..

[40] Ding Y., Koutrakis P. (2000). Development of a dichotomous slit nozzle virtual impactor. J. Aerosol Sci..

[41] Loo B.W., Cork C.P. (1988). Development of high efficiency virtual impactors. Aerosol Sci. Technol..

[42] Chen B.T., Yeh H.C. (1987). An improved virtual impactor: Design and performance. J. Aerosol Sci..

[43] Jurcik B., Wang H.C. (1995). On the shape of impactor efficiency curves. J. Aerosol Sci..

